# Apigenin Ameliorates Dyslipidemia, Hepatic Steatosis and Insulin Resistance by Modulating Metabolic and Transcriptional Profiles in the Liver of High-Fat Diet-Induced Obese Mice

**DOI:** 10.3390/nu8050305

**Published:** 2016-05-19

**Authors:** Un Ju Jung, Yun-Young Cho, Myung-Sook Choi

**Affiliations:** 1 Department of Food Science and Nutrition, Pukyong National University, 45 Yongso-ro, Nam-gu, Busan 48513, Korea; jungunju@pknu.ac.kr; 2 Biotech Research Center, AMICOGEN INC., 64, Dongbu-ro 1259 beon-gil, Jinseong-myeon, Jinju-si, Gyeongsangnamdo 660-852, Korea; yycho@amicogen.com; 3 Department of Food Science and Nutrition, Kyungpook National University, 1370 Sankyuk Dong Puk-ku, Daegu 702-701, Korea

**Keywords:** apigenin, hepatic metabolic and transcriptional responses, hepatic steatosis, high-fat diet-induced obesity, insulin resistance

## Abstract

Several *in vitro* and *in vivo* studies have reported the anti-inflammatory, anti-diabetic and anti-obesity effects of the flavonoid apigenin. However, the long-term supplementary effects of low-dose apigenin on obesity are unclear. Therefore, we investigated the protective effects of apigenin against obesity and related metabolic disturbances by exploring the metabolic and transcriptional responses in high-fat diet (HFD)-induced obese mice. C57BL/6J mice were fed an HFD or apigenin (0.005%, *w*/*w*)-supplemented HFD for 16 weeks. In HFD-fed mice, apigenin lowered plasma levels of free fatty acid, total cholesterol, apolipoprotein B and hepatic dysfunction markers and ameliorated hepatic steatosis and hepatomegaly, without altering food intake and adiposity. These effects were partly attributed to upregulated expression of genes regulating fatty acid oxidation, tricarboxylic acid cycle, oxidative phosphorylation, electron transport chain and cholesterol homeostasis, downregulated expression of lipolytic and lipogenic genes and decreased activities of enzymes responsible for triglyceride and cholesterol ester synthesis in the liver. Moreover, apigenin lowered plasma levels of pro-inflammatory mediators and fasting blood glucose. The anti-hyperglycemic effect of apigenin appeared to be related to decreased insulin resistance, hyperinsulinemia and hepatic gluconeogenic enzymes activities. Thus, apigenin can ameliorate HFD-induced comorbidities via metabolic and transcriptional modulations in the liver.

## 1. Introduction

The global prevalence of obesity and associated metabolic complications has increased in recent decades [1]. Obesity, especially abdominal obesity, is a main risk factor and a feature of metabolic syndrome, including insulin resistance, type 2 diabetes, dyslipidemia and nonalcoholic fatty liver disease (NAFLD) [2]. Evidence has suggested that inflammation, a hallmark of metabolic syndrome, can trigger obesity and obesity-related metabolic diseases [2,3]. Therefore, the use of anti-inflammatory phytochemicals is one of the strategies for treating obesity and its associated metabolic disturbances [4]. Moreover, obesity is directly associated with NAFLD, which includes a spectrum of disease ranging from simple hepatic steatosis to non-alcoholic steatohepatitis [5]. Excessive hepatic lipid accumulation is associated with systemic and hepatic inflammation, as well as dysregulated lipid metabolism [6]. Some herbal products, including silymarin, a lipophilic extract derived from milk thistle, have been reported to exhibit beneficial effects in NAFLD [7].

Flavonoids are a group of phytochemicals present in various fruits and vegetables. Among the diverse flavonoids, apigenin (5,7-dihydroxy-2-(4-hydroxyphenyl)-4H-1-benzopyran-4-one) is a flavone common in chamomile, parsley, onions, grapefruit and oranges [8]. It has been shown to reduce inflammation and has beneficial effects against cardiovascular disease and cancer [8]. In addition, recent studies have demonstrated the anti-obesity and anti-diabetic effects of apigenin. It suppressed adipogenesis in 3T3-L1 cells by activating 5′ AMP-activated protein kinase (AMPK) and by inhibiting mitotic clonal expansion [9,10]. It also improved glucose homeostasis, glucose tolerance and hepatic lipid metabolism in mice fed a high-fat diet (HFD) [11]. Moreover, apigenin (0.05%, *w*/*w*) slightly reduced food intake and body weight gain for 30 days in HFD-induced obese mice [12]. However, little is known about the long-term supplementary effects of low-dose apigenin on obesity and its associated metabolic disturbances, as well as the molecular mechanisms underlying its actions. Here, we examined the effects of apigenin (0.005%, *w*/*w*) on adiposity, insulin resistance, dyslipidemia and NAFLD in mice fed an HFD for 16 weeks and elucidated the metabolic and transcriptional mechanisms involved.

## 2. Materials and Methods

### 2.1. Animals

Four-week-old male C57BL/6J mice (*n* = 24) were purchased from the Jackson Laboratory (Bar Harbor, ME, USA). All mice were individually housed under constant temperature (24 °C) with a 12-h light/dark cycle, fed the AIN-76 (AIN, the American Institute of Nutrition) semi-purified diet for 1 week after arrival and then randomly divided into two groups. The mice were fed an HFD consisting of 20% (*w*/*w*) fat and 1% (*w*/*w*) cholesterol (*n* = 12) or HFD with 0.005% (*w*/*w*) apigenin (Sigma Chemical, St. Louis, MO, USA, from parsley powder) (*n* = 12) for 16 weeks. The HFD contains 40 kcal% fat, 17 kcal% protein and 43 kcal% carbohydrate. In the HFD, 85% (*w*/*w*) of total fat was from lard, which contains high amounts of saturated fat, and 15% (*w*/*w*) of total fat was from soybean oil, an unsaturated fat source. They were provided free access to food and distilled water, and food consumption, body weight and fasting blood glucose levels were measured daily, weekly and every 2 weeks. At the end of the experimental period, all mice were anesthetized with ether after a 12-h fast, and blood was taken from the inferior vena cava for the determination of plasma parameters. The liver and adipose tissue were removed, rinsed with physiological saline, weighed, immediately frozen in liquid nitrogen and stored at −70 °C until analysis. Studies were performed using protocols for animal studies approved by the Ethics Committee at Kyungpook National University (KNU-2010-4-14).

### 2.2. Levels of Fasting Blood Glucose, Plasma Insulin and Homeostatic Index of Insulin Resistance

Every 2 weeks, 12-h fasting whole blood samples were obtained from the tail veins, and the fasting blood glucose concentration was measured using a glucose analyzer (GlucDr supersensor, Allmedicus, Korea). The plasma insulin level was determined using a commercial radioimmunometric assay (MilliplexTM MAP Mouse endocrine kit, Millipore, Billerica, MA, USA), and the homeostatic index of insulin resistance (HOMA-IR) was calculated as follows: HOMA-IR = (fasting glucose (mmol/L) × fasting insulin (µL·U/mL))/22.51.

### 2.3. Plasma Adipocytokines, Lipids, Apolipoproteins and Aminotransferases Levels

Plasma levels of adipocytokines (leptin, monocyte chemoattractant protein-1 (MCP-1), interferon gamma-γ (IFN-γ), tumor necrosis factor-α (TNF-α) and interleukin-6 (IL-6)) were determined with a multiplex detection kit from Bio-Rad (Hercules, CA, USA). Plasma triglyceride, total cholesterol, HDL-cholesterol, alanine aminotransferase (ALT) and aspartate aminotransferase (AST) levels were measured using enzymatic assay kits (Asan Pharm, Seoul, Korea). Plasma free fatty acid (FFA, Wako, Tokyo, Japan), apolipoprotein A1 (apoA1, Eiken, Tokyo, Japan) and apolipoprotein B (apoB, Eiken, Tokyo, Japan) levels were also determined using enzymatic kits. The ratio of HDL cholesterol to total cholesterol (HTR) and the atherogenic index (AI) were calculated using the following Equations (1) and (2):

HTR = ((HDL-cholesterol)/(total cholesterol)) × 100(1)

AI = ((total cholesterol) − (HDL-cholesterol)/(HDL-cholesterol))(2)

### 2.4. Morphology of Liver

The livers were removed from each mouse, fixed in 10% (*v*/*v*) paraformaldehyde/phosphate-buffered saline (PBS) and then embedded in paraffin for staining with hematoxylin and eosin. The stained areas were viewed using an optical microscope (Nikon, Tokyo, Japan) with a magnifying power of ×200.

### 2.5. Hepatic Enzymes Activity

Hepatic cytosolic, mitochondrial and microsomal fractions were prepared as previously described [9]. The activities of cytosolic glucokinase, phosphoenolpyruvate carboxykinase (PEPCK), mitochondrial glucose-6-phosphatase (G6Pase), microsomal phosphatidate phosphohydrolase (PAP), acyl CoA cholesterol acyltransferase (ACAT) and glycogen content were measured according to previously described procedures [13,14].

### 2.6. RNA Preparation

Total RNA was extracted from the liver using TRIzol RIZOL reagent (Invitrogen, Grand Island, NY, USA), and the RNA purity and integrity were evaluated by microfluidics analysis using the Agilent 2100 Bioanalyzer (Agilent Technologies, Santa Clara, CA, USA). RNA samples were then stored at −70 °C prior to further analysis by microarray and real-time quantitative PCR (RT-qPCR). To reduce variation among individuals within each of the two groups, total RNA from mice of the same group was pooled together in equal amounts to generate a mixed sample. These three pooled RNA sample sets were subsequently used for RT-qPCR and microarray analysis.

### 2.7. Microarray Analysis and RT-qPCR

Biotinylated cRNA was generated using the Ambion Illumina RNA amplification kit (Ambion, Waltham, MA, USA). A total of 750 ng biotinylated cRNA per sample was hybridized to Illumina MouseWG-6 v2 Expression BeadChips (Illumina, San Diego, CA, USA) for 16–18 h at 58 °C, and hybridized arrays were washed and stained with Amersham fluorolink streptavidin-Cy3 (GE Healthcare Bio-Sciences, Little Chalfont, U.K.) following the standard protocol in the bead array manual. BeadChips were then scanned using an Illumina BeadArray Reader, and raw gene expression data were obtained from the array scanned images using the Illumina BeadStudio software. Probe signal intensities were quantile normalized and log transformed. Limma was used to determine significantly differentially-expressed genes based on a false discovery rate of less than 5%, a Benjamin and Hochberg-adjusted *p*-value of less than 0.05 and a fold change greater than 1 [15]. The DAVID (Database for Annotation, Visualization and Integrated Discovery) Functional Annotation Tool was used to identify enriched biological themes and to cluster redundant annotation terms.

For the validation of microarray data, several randomly-selected genes (Lpl, Pparγ, Srebf1, Dgat2, Scd1 and Cidea) were measured independently by RT-qPCR using the same pooled RNA samples that were hybridized to BeadChips. Total RNA (1 μg) was reverse-transcribed into cDNA using the QuantiTect^®^ reverse transcription kit (Qiagen, Hilden, Germany), and then, mRNA expression was quantified by RT-qPCR using the SYBR green PCR kit (Qiagen, Hilden, Germany) and the CFX96TM real-time system (Bio-Rad, Hercules, CA, USA). Cycle thresholds were determined based on SYBR green emission intensity during the exponential phase. Ct data were normalized using Gapdh, and relative gene expression was calculated with the 2^−ΔΔ*C*t^ method.

### 2.8. Statistical Analysis

The values were expressed as the means ± standard error (S.E). Significant differences between two groups were determined by Student’s *t*-test or the Wilcoxon *t*-test using the SPSS program (SPSS Inc., Chicago, IL, USA). Results were considered statistically significant at *p* < 0.05.

## 3. Results

### 3.1. Apigenin Did Not Alter Food Intake, Body Weight Gain and Fat Accumulation

**Figure 1:**
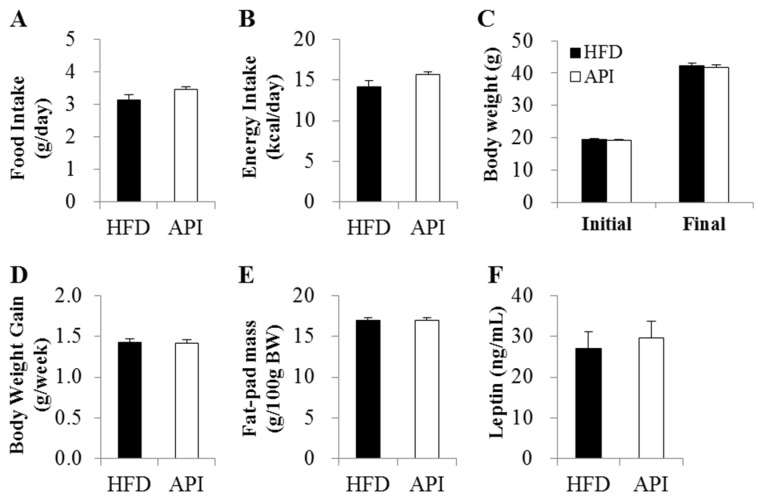
Effects of apigenin on (**A**) food intake; (**B**) energy intake; (**C**,**D**) body weight; (**E**) fat-pad mass and (**F**) plasma leptin level in C57BL/6J mice fed a high-fat diet. Data are shown as the means ± S.E. HFD: high-fat diet (20% fat, 1% cholesterol); API: HFD + 0.005% apigenin.

Supplementation of apigenin did not alter food and energy intake in mice (Figure 1A,B). Initial body weight, final body weight, body weight gain and fat mass also did not differ between the two groups (Figure 1C–E). Moreover, apigenin did not affect circulating levels of leptin, a representative adipokine secreted from adipocytes in proportion to fat mass and involved in the regulation of food intake and energy homeostasis [2].

### 3.2. Apigenin Decreased Fasting Blood Glucose and Plasma Insulin Levels and Ameliorated Insulin Resistance and Inflammation

**Figure 2:**
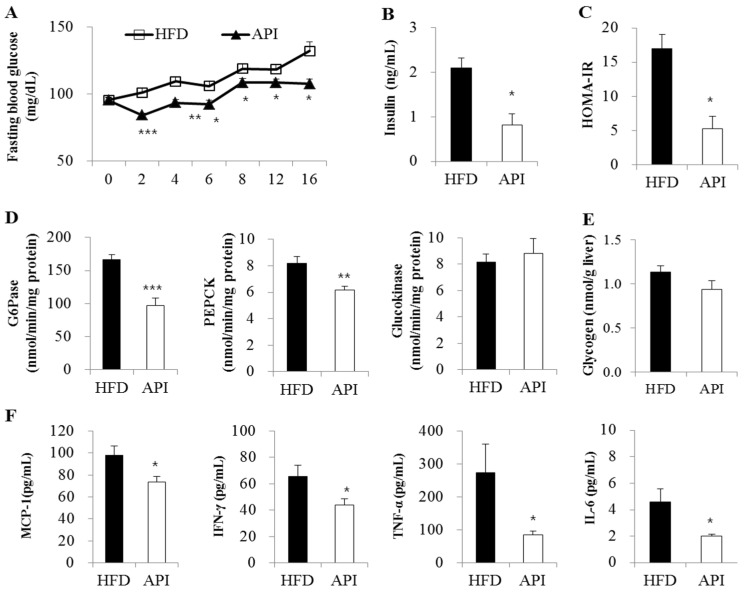
Effects of apigenin on (**A**) fasting blood glucose level; (**B**) plasma insulin level; (**C**) homeostatic index of insulin resistance (HOMO-IR); (**D**) hepatic glucose metabolism-related enzyme activities; (**E**) hepatic glycogen content and (**F**) plasma pro-inflammatory marker levels in C57BL/6J mice fed a high-fat diet. Data are shown as the means ± S.E. Values are significantly different between the high-fat diet and apigenin groups according to Student’s *t*-test: * *p* < 0.05; ** *p* < 0.01; *** *p* < 0.001. HFD: high-fat diet (20% fat, 1% cholesterol); API: HFD + 0.005% apigenin.

We next examined whether apigenin influenced HFD-induced insulin resistance and inflammation. Apigenin significantly decreased fasting blood glucose levels after two weeks of supplementation in HFD-fed mice (Figure 2A). Levels of plasma insulin and HOMA-IR, a method used to quantify insulin resistance and β-cell function [16], were also significantly decreased by apigenin (Figure 2B,C). Although hepatic glucokinase activity and glycogen content remained unaffected (Figure 2D,E), hepatic PEPCK and G6Pase activities were decreased in apigenin-supplemented mice. Moreover, plasma levels of pro-inflammatory mediators, such as MCP-1, IFN-γ, TNF-α and IL-6, were significantly decreased by apigenin (Figure 2F).

### 3.3. Apigenin Improved Dyslipidemia, Hepatic Steatosis and Hepatomegaly

**Figure 3:**
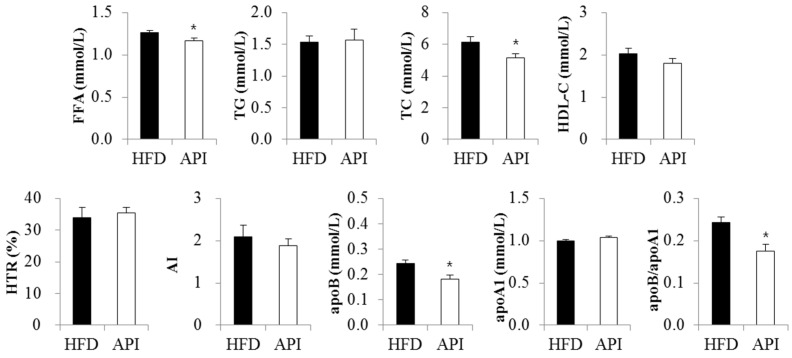
Effect of apigenin on plasma lipids and apolipoproteins in C57BL/6J mice fed a high-fat diet. Data are shown as the means ± S.E. Values are significantly different between the high-fat diet and apigenin groups according to Student’s *t*-test: * *p* < 0.05. HFD: high-fat diet (20% fat, 1% cholesterol); API: HFD + 0.005% apigenin.

There were no significant differences in plasma triglyceride, HDL-cholesterol and apoA1 levels between the two groups (Figure 3). However, plasma free fatty acid levels were significantly decreased in apigenin-supplemented obese mice. Plasma total-cholesterol levels, as well as plasma apoB levels and the apoB/apoA1 ratio were also markedly decreased by apigenin.

**Figure 4:**
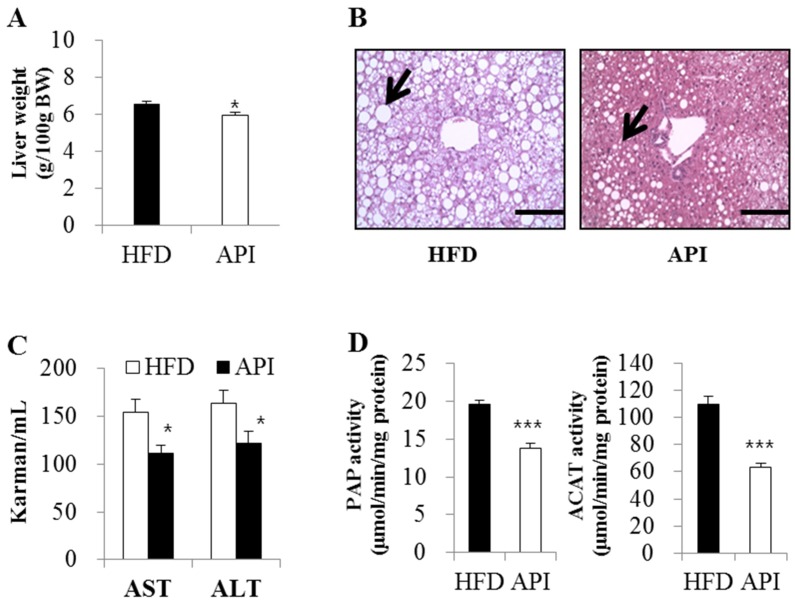
Effect of apigenin on liver weight (**A**), hepatic morphology (**B**), plasma transaminases activities (**C**) and activities of hepatic enzymes controlling the synthesis of triglyceride and cholesterol ester (**D**) in C57BL/6J mice fed a high-fat diet; ((**A**), (**C**), and (**D**)) Data are shown as the means ± S.E. Values are significantly different between the high-fat diet and apigenin groups according to Student’s *t*-test: * *p* < 0.05, ** *p* < 0.05; (**B**) Original magnification ×200. Bar, 50 M. HFD: high-fat diet (20% fat, 1% cholesterol); API: HFD + 0.005% apigenin.

In addition, apigenin significantly decreased liver weight and hepatic lipid droplets’ accumulation along with plasma ALT and AST levels (Figure 4A–C). Hepatic PAP and ACAT activities were also significantly lowered in apigenin-supplemented obese mice (Figure 4D).

### 3.4. Liver Gene Expression Profiles in Response to Apigenin

**Figure 5:**
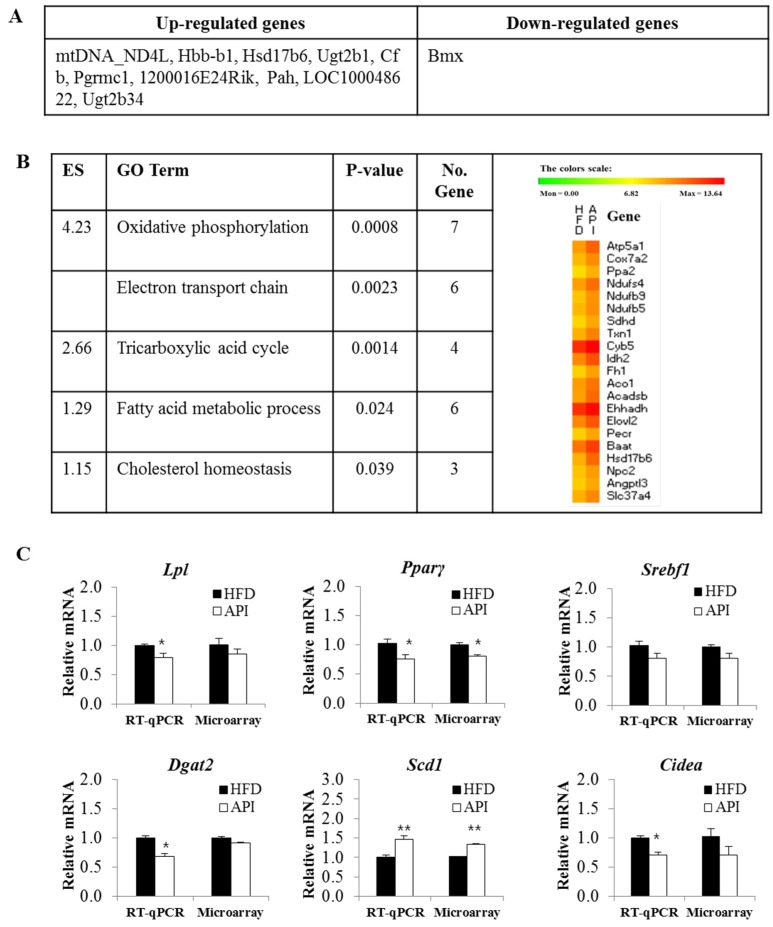
The top 10 most upregulated and downregulated genes in the livers of the apigenin group compared to the control group (**A**); functional gene ontologies associated with the apigenin-responsive genes (**B**); and real-time quantitative PCR validation (**C**); (**A**,**B**) comparison of differentially-expressed genes in the apigenin group vs. the control group using Benjamin–Hochberg adjusted *p*-value < 0.05, FDR (False Discovery Rate) <5%, fold change >1; (**B**) functional gene ontology terms enriched among apigenin responsive genes are clustered according to biological processes (enrichment score >1) using DAVID. The heatmap shows the expression profiles of the representative apigenin responsive genes in each cluster; (**C**) Data are shown as the means ± S.E. Values are significantly different between the high-fat diet and apigenin groups according to Student’s *t*-test: * *p* < 0.05; ** *p* < 0.05. Microarray data based on pooled RNA hybridized to Illumina MouseWG-6 v2.0 BeadChips. HFD: high-fat diet (20% fat, 1% cholesterol); API: HFD + 0.005% Apigenin; Lpl: lipoprotein lipase; Pparγ: peroxisome proliferator-activated receptor γ; Srebf1: sterol regulatory element-binding transcription factor 1; Dgat2: diacylglycerol O-acyltransferase 2; Scd1: stearoyl-CoA desaturase-1; Cidea: cell death activator; ES: Enrichment Score.

To investigate changes in hepatic gene expression profiles in response to apigenin, we identified differentially-expressed genes in apigenin-supplemented mice compared to HFD control mice using microarray analysis. Of the 281 differentially-expressed genes in the two groups, 271 genes were upregulated, and one gene was downregulated. The top 10 differentially-upregulated genes and one downregulated gene are shown in Figure 5A. Functional annotation clustering using DAVID revealed that the majority of hepatic genes regulated by apigenin in HFD-fed mice were related to oxidative phosphorylation (OXPHOS), the electron transport chain, the tricarboxylic acid (TCA) cycle, fatty acid metabolism and cholesterol homeostasis (Figure 5B).

To further validate the reliability of our microarray data, we performed RT-qPCR on six randomly-selected genes (Lpl, Pparγ, Srebf1, Dgat2, Scd1 and Cidea). The results were in agreement with the microarray data, and RT-qPCR was more sensitive to small changes in gene expression compared to the microarray, similar to a previous study [15]. Except for Scd1, mRNA expression of lipolysis- and lipogenesis-related genes (Lpl, Pparγ, Srebf1, Dgat2 and Cidea) was downregulated in the livers of apigenin-supplemented mice (Figure 5C).

## 4. Discussion

In the present study, supplementation of an HFD with apigenin (0.005%, *w*/*w*) for 16 weeks did not alter food intake, body weight gain and fat accumulation in mice. These findings were in disagreement with previous findings, which demonstrated that apigenin (0.05%, *w*/*w*) reduced food intake and body weight gain for 30 days in HFD-fed mice [5], and it inhibited adipogenesis in 3T3-L1 cells through downregulation of PPARγ by activating AMPK and the modulation of mitotic clonal expansion [9,10]. The low-dose (0.005%, *w*/*w*) of apigenin used in the present study may be insufficient for suppressing food intake, body weight gain and body fat accumulation.

Insulin resistance and hyperinsulinemia are hallmarks of obesity and can induce many of the abnormalities associated with metabolic syndrome [17]. A previous study from our laboratory demonstrated that an HFD for 16 weeks led to increased fasting blood glucose, hyperinsulinemia and insulin resistance in mice compared to a normal diet [15]. In the present study, apigenin significantly decreased levels of fasting blood glucose, plasma insulin and HOMA-IR, a surrogate marker for insulin resistance, in HFD-fed mice. Moreover, it significantly decreased the activities of hepatic PEPCK and G6Pase. These are key enzymes for gluconeogenesis, and controlling hepatic gluconeogenesis is crucial for maintaining glucose homeostasis [18]. Insulin is the most important hormone, which inhibits gluconeogenesis, and it directly suppresses the transcription and activity of hepatic gluconeogenic enzymes [18]. Hepatic insulin resistance results in impaired insulin-induced suppression of gluconeogenesis in obese subjects, and gluconeogenesis closely correlates with the severity of diabetes and the degree of obesity [19]. Therefore, apigenin seems to decrease fasting blood glucose by inhibiting hepatic gluconeogenic enzyme activities, and the changes in hepatic glucose-regulating enzymes may be partly attributed to the apigenin-induced improvements in insulin resistance. Our findings were supported by a previous *in vitro* study that demonstrated the inhibitory effects of apigenin on gene expression of PEPCK and G6Pase in HepG2 cells [20]. Moreover, a significant decrease in fasting blood glucose levels after apigenin consumption was observed in HFD-fed mice [11] and streptozotocin-induced type 1 diabetic rats [21].

Inflammation is one of the main mechanisms of impaired insulin action. The production of adipocytokines is related to ectopic fat accumulation and decreases insulin sensitivity directly and/or indirectly in adipose tissue and the liver, leading to impaired glucose homeostasis [3,22,23]. For example, TNF-α is an important mediator of insulin resistance in obesity owing to its inhibitory effects on insulin receptor signaling. IL-6 also plays a direct role in insulin resistance by inhibiting insulin receptor signal transduction and insulin action in hepatocytes [24]. Their circulating levels were increased in insulin resistance states, such as obesity and type 2 diabetes, while they were decreased in response to weight reduction or anti-diabetic drugs [2,25,26]. IFN-γ is another pro-inflammatory cytokine mainly produced by T-cells, and it regulates insulin resistance in obesity by inducing pro-inflammatory cytokine expression in macrophages [27]. The production of IFN-γ by T-cells in adipose tissue was higher in HFD-induced obese mice than lean mice, and obese IFN-γ-deficient mice showed modest increases in insulin sensitivity [27]. O’Rourke *et al.* [28] also demonstrated a role for IFN-γ in the regulation of inflammation and glucose homeostasis in obesity though multiple mechanisms, including its effects on pro-inflammatory cytokine expression and macrophage phenotype. In addition to cytokines, obese adipose tissue secretes chemokines, such as MCP-1, which contribute to obesity-related insulin resistance [2]. A previous study reported that apigenin exerted anti-inflammatory activity *in vitro* and *in vivo* by inactivating NF-κB, thereby reducing the production of inflammatory mediators [29]. Since we also observed that plasma levels of pro-inflammatory mediators, such as MCP-1, IFN-γ, TNF-α and IL-6, were significantly decreased by apigenin, these changes could be partly associated with the decreased fat accumulation, improved insulin resistance and glucose homeostasis in apigenin-supplemented obese mice.

It is well known that abnormal lipid metabolism in obesity is regarded as a major driving force for dyslipidemia and hepatic steatosis, and insulin resistance and inflammation play an important role in the development of dyslipidemia and hepatic steatosis [2]. In insulin resistance states, the increased lipolysis of stored triglycerides in adipose tissue promotes the production of fatty acids, and the elevation of circulating free fatty acid inhibits the anti-lipolytic action of insulin and favors increased uptake into the liver, leading to dyslipidemia and hepatic steatosis. Free fatty acid can also serve as an endogenous signal to stimulate the production of pro-inflammatory mediators, such as TNF-α and IL-6, and inhibition of pro-inflammatory signaling pathways can prevent free fatty acid-induced insulin resistance [2,30]. Moreover, pro-inflammatory cytokines, including TNF-α and IL-6, increase circulating levels of total cholesterol, LDL-cholesterol and free fatty acid and stimulate over secretion of apoB in the liver [2]. In addition, elevated levels of TNF-α, IL-6 and MCP-1 were associated with the development of NAFLD, and pharmacological and genetic inhibition of these pro-inflammatory mediators ameliorated hepatic steatosis in obese animals [2,31].

In the present study, apigenin markedly decreased plasma total-cholesterol levels, as well as plasma apoB levels and the apoB/apoA1 ratio, indicating its protective role against atherogenic dyslipidemia in HFD-induced obese mice. In addition, plasma free fatty acid levels were significantly decreased in apigenin-supplemented obese mice. As described in the preceding text, increased delivery and uptake of free fatty acids into the liver promote the production of triglycerides, which ultimately leads to hepatic steatosis [2]. Moreover, increased levels of circulating free fatty acids, rather than excessive hepatic lipid accumulation, serve as an indicator of the degree of liver damage [32]. The plasma levels of ALT and AST, useful biomarkers of liver injury, as well as hepatic lipid droplet accumulation were elevated in HFD-induced obese animals [33]. In the present study, apigenin decreased hepatomegaly and hepatic lipid droplets’ accumulation along with plasma ALT and AST levels, indicating its beneficial effects on NAFLD.

Lipid droplets are dynamic organelles that govern the storage and turnover of lipids and comprise a core of storage of neutral lipids, *i.e.*, triglycerides and cholesterol esters. PAP is a rate-limiting enzyme for hepatic triglyceride synthesis, and the activity and expression of the lipin-1 gene encoding PAP were increased in HFD-fed obese mice or *ob/ob* mice [15,34], while deficiency of lipin-1 attenuated hepatic steatosis [34]. The esterified cholesterol is also a major component of lipid droplets, and ACAT, a key cholesterol-regulating enzyme involved in the hepatic esterification of cholesterol, facilitates cholesterol ester incorporation into lipid droplets [35]. The activity of ACAT in the livers of HFD-induced obese mice was higher than that of non-obese mice [15], and inhibition of ACAT improved abnormal lipid metabolism and hepatic steatosis in obese mice [36]. Alger *et al.* [37] also demonstrated that the increased accumulation of cholesterol ester in lipid droplets could limit the mobilization of hepatic triglycerides and decrease the release of very-low-density lipoprotein-triglyceride by the liver, leading to cholesterol-associated hepatic steatosis. Interestingly, we observed that hepatic PAP and ACAT activities were significantly lowered by apigenin. Based on these results, apigenin seemed to ameliorate hepatic steatosis by reducing lipid droplet accumulation via a decrease in plasma free fatty acid and an inhibition of hepatic enzyme activities involved in the synthesis of triglycerides and cholesterol esters in HFD-fed mice. Moreover, inhibition of hepatic ACAT may be a possible mechanism for decreased plasma total cholesterol and apoB levels observed in apigenin-supplemented mice, since ACAT inhibitors stimulate bile acid synthesis and inhibit apoB secretion, thus controlling plasma cholesterol levels, as well as hepatic cholesterol biosynthesis and catabolism [38,39].

In aerobic organisms from bacteria to humans, OXPHOS is the major source of energy-rich ATP, and the electron transport chain is responsible for creating the proton gradient that drives the generation of ATP via OXPHOS. The electron transport chain is composed of four protein complexes (complexes I, II, III and IV), which are found in the inner membrane of a mitochondrion. Ndufs4 is a gene encoding the NADH-ubiquinone oxidoreductase subunit of complex I, the first multisubunit enzyme complex of the mitochondrial respiratory chain, which plays a critical role in cellular generation of ATP. In a recent study, a deletion of Ndufs4 led to an inhibition of OXPHOS and increases in circulating free fatty acid and inflammation [40]. In addition to Atp5a1, which encodes a subunit of mitochondrial ATP synthase (complex V), other mitochondrial complex I and II subunit-encoding genes (e.g., Ndufb5, Ndufb9 and Sdhd) were downregulated in obese subjects and type 2 diabetic patients, and impaired mitochondrial OXPHOS was proposed as an etiological mechanism underlying insulin resistance [41]. Interestingly, apigenin upregulated nine genes involved in OXPHOS and the electron transport chain, including Atp5a1, Cox7a2, Ndufs4, Ndufb5, Ndufb9 and Sdhd.

We also observed that TCA cycle genes (Idh2, Fh1, Aco1) and fatty acid oxidation genes (Acadsb, Ehhadh) were upregulated in the livers of apigenin-supplemented obese mice, along with other genes that facilitate fatty acid utilization (e.g., Elovl2 and Pecr) (Figure 5B). Fatty acid oxidation is the catabolic process by which fatty acid molecules are broken down in the mitochondria to produce acetyl coenzyme A, which enters the TCA cycle [42]. The reactions of the TCA cycle generate NADH and FADH2, which are in turn used by the OXPHOS pathway to generate ATP [42]. The mRNA expression of Elovl2, which is involved in the elongation required for the synthesis of docosahexaenoic acid, was reduced in the livers of obese subjects, and an intervention to improve hepatic steatosis and diabetes upregulated hepatic Elovl2 expression along with hepatic fatty acid oxidation [43,44]. Since the synthesis of docosahexaenoic acid from linolenic acid requires one round of peroxisomal β-oxidation in addition to elongation and desaturation [45], the observed upregulation of Elovl2 may be related to the upregulated expression of peroxisomal fatty acid oxidation genes, such as Ehhadh. Hepatic gene expression of Pecr, another fatty acid elongation gene, was also upregulated by a dietary strategy for the treatment of hepatic steatosis [46], and its gene expression was downregulated in mice after acute inhibition of β-oxidation [47]. Taken together, our microarray data suggest that apigenin can upregulate the expression of hepatic genes involved in energy metabolism, such as OXPHOS, electron transport chain, TCA cycle and fatty acid oxidation, which may contribute to improved metabolic abnormalities, such as hepatic steatosis, dyslipidemia and insulin resistance.

The microarray data of the liver also revealed that supplementation of the HFD with apigenin upregulated the expression of genes involved in cholesterol homeostasis. The liver plays a critical role in cholesterol metabolism and in controlling its removal through the bile. Along with cholesterol esterification, the hepatic conversion of cholesterol into bile acid is an important cholesterol-metabolizing pathway for the elimination of excess cholesterol. Approximately 95% of bile acids are reabsorbed in the intestine and transported back to the liver, and the remaining 5% is lost via the feces and compensated via *de novo* bile acid synthesis from cholesterol in the liver. Prior to bile acid secretion, the synthesized bile acids in the liver need to be conjugated to either glycine or taurine to form bile salts. The bile acid conjugation processes can protect against hepatic damage, because glycine/taurine conjugates are generally less toxic and more hydrophilic than the primary bile acids [48]. Baat is implicated in the conjugation of *de novo*-synthesized bile acids from cholesterol in the liver. Defects in Baat can cause intrahepatic cholestasis, which occurs in a subgroup of patients with NAFLD [49]. Similar to our study, hepatic Baat mRNA expression was upregulated by treatment with an agent protecting against diet-induced NAFLD [50]. Moreover, we observed that apigenin upregulated hepatic gene expression of NPC2 encoding a cholesterol-binding protein, Niemann-Pick type C 2, which positively regulates biliary cholesterol secretion via stimulation of ABCG5/ABCG8-mediated cholesterol efflux [51]. Since the amount of cholesterol secreted into the bile each day is comparable to the amounts synthesized in the liver and absorbed from the intestine, the regulation of biliary cholesterol secretion by Npc2 is thought to be important in the maintenance of the whole-body cholesterol level. Moreover, lack of Npc2 increased cellular cholesterol, suggesting the role for Npc2 in the regulation of sterol homeostasis [52].

In addition to bile acid, steroid hormones are generally synthesized from cholesterol. Among the various genes involved in steroid hormone synthesis, the gene expression of Hsd17b6, which is involved in the conversion of testosterone back to androstenedione, was upregulated in the livers of apigenin-supplemented obese mice. The Hsd17b6 is suggested to act as a modifier gene influencing insulin resistance and obesity [53], and obese subjects show lower Hsd17b6 gene expression in the liver [43], whereas its gene expression is increased in the livers of obesity-resistant animals [54]. In the present study, apigenin also upregulated mRNA expression of hepatic Slc37a4 mRNA, whose expression was decreased during the progression of liver fibrosis [55], and suppression of this molecule was associated with stimulation of *de novo* lipogenesis and the development of hepatic steatosis [56]. A recent study suggested that it is involved in processes determining the total plasma cholesterol concentration [57]. In addition, we observed that apigenin increased hepatic gene expression of angiopoietin-like (Angptl) 3, which is exclusively expressed in the liver and is involved in the trafficking and metabolism of lipids. Although genetic deletion of Angptl3 was associated with reduced circulating HDL-cholesterol and triglyceride levels in mice [58], apigenin did not affect these circulating lipid levels in the present study. This may be related to unchanged hepatic Angptl8 mRNA expression, because Quagliarini *et al.* [59] recently demonstrated that the plasma triglyceride level was not altered in mice expressing Angptl3 alone, but coexpression of Angptl8 leads to hypertriglyceridemia despite a reduction in the circulating Angptl3 level. They have suggested that Angptl8 is a paralog of Angptl3, which controls triglyceride metabolism together with Angptl3.

We also observed that mRNA expression of lipolysis- and lipogenesis-related genes (Lpl, Pparγ, Srebf1, Dgat2 and Cidea) was downregulated in the livers of apigenin-supplemented mice. The gene expression of Lpl, which controls fatty acid uptake through the hydrolysis of triglyceride-rich lipoproteins, was higher in obese subjects with NAFLD compared to subjects without NAFLD [60], and liver-specific overexpression of Lpl induced hepatic steatosis and insulin resistance in mice [61]. Therefore, it is proposed that upregulation of Lpl can contribute to hepatic steatosis by promoting the incorporation of circulating fatty acids into intrahepatic triglycerides [62]. Similar to Pparγ, Srebf1, Dgat2 and Cidea, several lipogenic genes have also been suggested as steatogenic factors in the liver [62]. The hepatic overexpression of Dgat2, which catalyzes the final step of triglyceride synthesis, led to hepatic steatosis in mice [63], and liver-specific disruption of Pparγ or Srebf1 protected mice against hepatic steatosis [64]. Moreover, Cidea promoted large lipid droplets’ accumulation in the liver [65]. In contrast, a deficiency of hepatic Scd1, which converts saturated fatty acids to monounsaturated fatty acids, provided protection against hepatic steatosis induced by a high-carbohydrate diet, but not HFD [66]. Together, our data indicate that the molecular mechanism of apigenin action involves not only activation of energy metabolism and regulation of cholesterol metabolism, but also reduction of lipolysis and lipogenesis in the liver.

## 5. Conclusions

**Figure 6:**
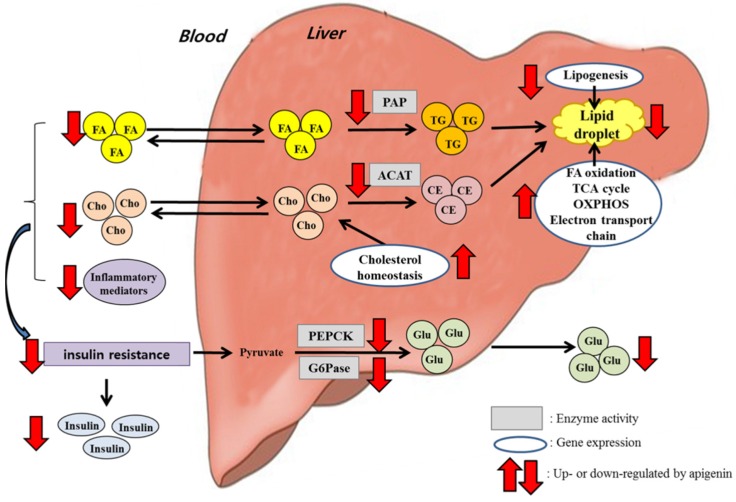
Schematic diagram showing the mechanisms underlying the beneficial effects of apigenin on obesity-related metabolic disturbances. Apigenin decreased the activities of hepatic enzymes controlling triglyceride synthesis and cholesterol esterification and increased the expression of hepatic genes involved in fatty acid oxidation, the TCA cycle, OXPHOS, the electron transport chain and cholesterol homeostasis while decreasing the expression of hepatic lipogenic and lipolytic genes, indicating that these changes may be potential mechanisms for improving dyslipidemia and hepatic steatosis in HFD-fed mice. Moreover, apigenin decreased plasma pro-inflammatory adipocytokines levels and hepatic gluconeogenic enzyme activities, which may be partly associated with the improved hyperglycemia, hyperinsulinemia and insulin resistance.

Long-term supplementation of apigenin (0.005%, *w*/*w*) to HFD-fed mice ameliorated dyslipidemia and hepatic steatosis. These beneficial effects were accompanied by decreased activities of hepatic enzymes controlling triglyceride synthesis and cholesterol esterification and by increased expression of hepatic genes involved in fatty acid oxidation, the TCA cycle, OXPHOS, the electron transport chain and cholesterol homeostasis, as well as decreased expression of hepatic lipogenic and lipolytic genes, as summarized in Figure 6. Furthermore, apigenin lowered levels of pro-inflammatory cytokines and chemokines in plasma, and it improved hyperglycemia, hyperinsulinemia and insulin resistance. The improved glucose metabolism by apigenin appeared to be mediated through the inhibition of hepatic gluconeogenic enzyme activities. Taken together, our findings suggest that apigenin may help to ameliorate HFD-induced metabolic disturbances, such as dyslipidemia, hepatic steatosis and insulin resistance.
